# CD109 Deletion Promotes Myofibroblast Differentiation and Smad-Dependent Matrix Accumulation in Skin Fibrosis

**DOI:** 10.3390/ijms27062834

**Published:** 2026-03-20

**Authors:** Liqin Xu, Setareh Garousi, Adel Batal, Kenneth W. Finnson, Anie Philip

**Affiliations:** 1 Division of Plastic Surgery, Department of Surgery, McGill University, Montreal, QC H3G 1A4, Canada; liqin.xu@mcgill.ca (L.X.); setareh.garousi@mail.mcgill.ca (S.G.); adel.batal@mail.mcgill.ca (A.B.); kenneth.finnson@mcgill.ca (K.W.F.); 2 The Research Institute of the McGill University Health Centre, Montreal, QC H4A 3J1, Canada; 3 Divisions of Dermatology and Experimental Medicine, Department of Medicine, McGill University, Montreal, QC H4A 3J1, Canada

**Keywords:** CD109, skin fibrosis, TGF-β signaling, scleroderma, mouse model

## Abstract

Skin fibrosis is characterized by excessive extracellular matrix (ECM) deposition, leading to tissue dysfunction and scarring. Transforming growth factor (TGF)-β is a central mediator of fibrosis. We previously identified CD109 as a TGF-β co-receptor and negative regulator of TGF-β signaling and fibrotic responses and showed that its epidermal overexpression reduces dermal fibrosis in vivo. However, the effects of CD109 loss in the dermis remain unclear. The current study investigates the impact of CD109 knockout (KO) on skin fibrosis using a bleomycin-induced fibrosis mouse model. Following bleomycin treatment, CD109 KO mice showed increased collagen I deposition and elevated fibronectin, CCN2, and α–smooth muscle actin expression in the skin, indicating enhanced ECM production and myofibroblast differentiation compared with wild-type mice. Additionally, CD109 KO mice displayed enhanced Smad1 and Smad2/3 phosphorylation in the skin, indicating heightened TGF-β signaling. In vitro, CD109 KO fibroblasts exhibited increased TGF-β-induced migration and collagen contraction. These findings suggest that CD109 deficiency exacerbates dermal fibrosis by promoting TGF-β/Smad signaling and myofibroblast activation. Given its dysregulation in fibrotic disorders such as scleroderma, our results identify CD109 as a key regulator of skin homeostasis by modulating ECM production and fibroblast activation, underscoring its potential as a therapeutic target in fibrotic disorders.

## 1. Introduction

Skin fibrosis, a condition marked by excessive accumulation of extracellular matrix (ECM) proteins like collagen and fibronectin, poses a substantial clinical challenge, especially in disorders such as scleroderma (systemic sclerosis, SSc) and hypertrophic scars [1,2]. This condition is closely associated with impaired wound healing processes and abnormal fibroblast function, which results in increased tissue stiffness, diminished functionality, and cosmetic issues [3]. Central to these processes is transforming growth factor beta (TGF-β), a potent pro-fibrotic cytokine that plays a pivotal role in wound healing and inflammatory responses [1,4].

TGF-β is a pleiotropic cytokine that plays fundamental roles in development and homeostasis by regulating cellular processes including proliferation, differentiation and ECM production [5]. TGF-β canonical signaling is transmitted by a pair of transmembrane serine/threonine kinases known as the TGF-β type II (TβRII) and type I (TβRI, also known as activin receptor-like kinase-5 or ALK5) receptors [6,7]. TGF-β first binds to TβRII, a constitutively active kinase, which in turn phosphorylates ALK5, resulting in its activation [6,7,8]. ALK5 then phosphorylates intracellular Smad2 and Smad3 proteins, which form a complex with Smad4, enter the nucleus and regulate gene expression [9,10]. TGF-β can also signal through a different type I receptor known as activin receptor-like kinase-1 (ALK1), which phosphorylates Smad-1protein [11,12,13,14], leading to different intracellular signaling outcomes including the inhibition of ALK5-Smad2/3 signaling [14,15]. Furthermore, TGF-β is also able to activate so-called ‘non-Smad’ pathways including the mitogen-activated protein (MAP) kinases, phosphatidylinositol-3-kinase and Rho-like GTPases [16,17,18]. The critical role of TGF-β signaling in the development of fibrosis through the activation of fibroblasts and the synthesis of ECM proteins is well documented [19,20].

CD109 is a glycosylphosphatidylinositol (GPI)-anchored protein and a member of the α2-macroglobulin (α2M)/C3, C4, C5 complement family of thioester-containing proteins [21,22,23]. We were the first to demonstrate that CD109 functions as a TGF-β co-receptor [21], negatively regulating TGF-β signaling and inhibiting responses such as extracellular matrix (ECM) synthesis and skin fibrosis [21,24,25,26,27,28,29]. We have shown that CD109 is a critical regulator of TGF-β receptor levels and signaling by promoting TGF-β receptor internalization and Smurf2-mediated degradation via the caveolar pathway [30,31,32]. We found that transgenic (TG) mice overexpressing CD109 in the epidermis exhibit reduced inflammatory cell (neutrophil and macrophage) infiltration, granulation tissue formation, and improved collagen fiber organization during wound healing, when compared to WT littermates [27]. We also showed that epidermal overexpression of CD109 leads to a decrease in bleomycin-induced skin fibrosis, as evidenced by a significant reduction in dermal thickness, collagen cross-linking, collagen and fibronectin content and phospho-Smad2/3 levels, as compared to their WT littermates [28]. Under basal conditions, the TG mice with epidermal CD109 overexpression exhibit decreased ALK5-Smad2/3 signaling and enhanced ALK1-Smad1/5 activation. The decreased ECM production in the dermis displayed by these mice was shown to be due to a paracrine mechanism that likely involves the secretion of soluble factors from epidermal keratinocytes acting on the underlying dermal fibroblasts [24].

The Global CD109 KO mice have been characterized by others previously and have been shown to display epidermal hyperplasia, transient impairment of hair growth and infiltration of immune cells (neutrophils, T and B lymphocytes) in the dermis, but no difference in skin wound closure rate was noted as compared to WT littermates [33]. Also, in a later study, this group has reported that these mice exhibit osteopenia with an osteoporosis-like phenotype in vivo [34]. Another study using these mice has shown that CD109 deletion suppresses chemically induced skin tumorigenesis [35]. Whether CD109 deficiency affects experimental skin fibrosis has not been investigated. Here, we show that CD109 KO mice display enhanced bleomycin-induced skin fibrosis with significantly increased collagen deposition, ECM production, and myofibroblast differentiation as compared to WT mice. Additionally, CD109 KO mice display enhanced phosphorylation of Smad2 and Smad3 proteins during bleomycin-induced skin fibrosis, indicating heightened TGF-β signaling. In vitro, CD109 KO fibroblasts exhibit increased basal proliferation, as well as TGF-β1-induced cell migration and collagen contraction compared to WT fibroblasts. These findings suggest that CD109 deficiency worsens dermal fibrosis by enhancing TGF-β/Smad signaling and myofibroblast activity, highlighting CD109 as an important regulator of skin homeostasis and a potential therapeutic target in fibrotic skin diseases like scleroderma.

## 2. Results

**CD109 KO mice display enhanced dermal collagen deposition as compared to WT mice, during bleomycin-induced skin fibrosis.**

**Figure 1:**
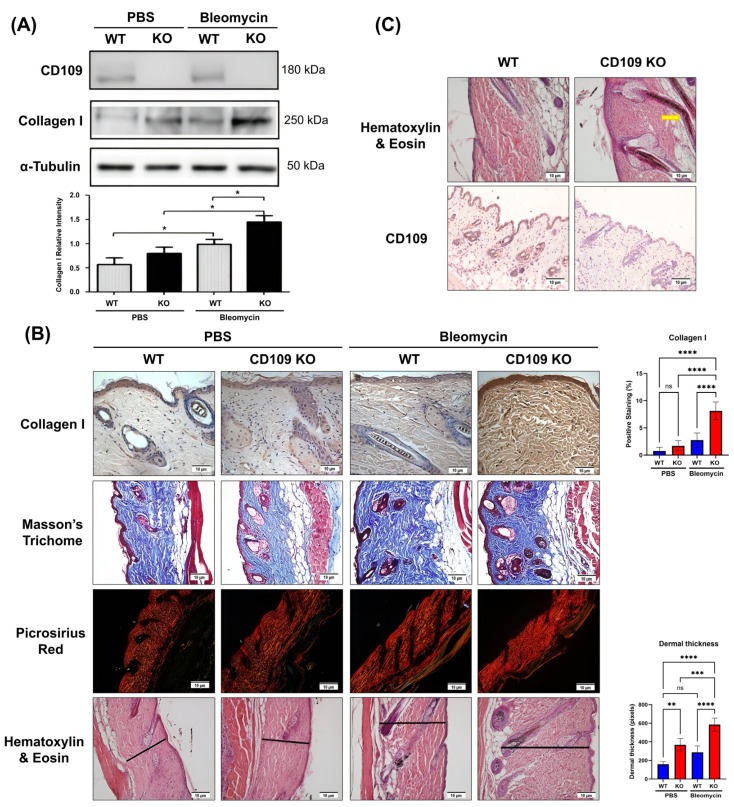
Increased dermal type I collagen production and deposition in CD109 KO mice skin following bleomycin injections. WT and CD109 KO mice received alternating PBS or bleomycin injections for 28 days, after which their skin was analyzed (n = 4 per condition). (**A**) Western blot analysis of collagen I levels in WT and CD109 KO mice treated with either PBS or bleomycin. Protein expression was quantified using ImageJ 1.54g and plotted with GraphPad Prism 10. Student’s *t*-test is used for the calculation of statistical significance. (**B**) Immunohistochemical, Masson’s Trichrome, Picrosirius Red, and Hematoxylin and Eosin staining in skin sections from WT and CD109 KO mice treated with PBS or bleomycin. Ordinary one-way ANOVA was used for the calculation of statistical significance. Black bar: Dermal thickness. (**C**) Hematoxylin and eosin and IHC staining for CD109 in skin samples from both WT and CD109 KO mice treated with intradermal PBS injections. A yellow arrow marks the kinked hair shaft. Ordinary one-way ANOVA was used for the calculation of statistical significance. WT: Wild-type, KO: Knockout, PBS: Phosphate-buffered saline. Scale bars: 10 µm. * *p* < 0.05, ** *p* < 0.01, *** *p* < 0.001, **** *p* < 0.0001; ns, not significant. The black line delineates the dermal region.

The levels of collagen 1 in skin tissue extracts of CD109 KO versus WT mice did not show significant differences under basal conditions. However, during bleomycin-induced skin fibrosis, CD109 KO mice exhibited a significantly greater increase in collagen I levels in their skin as compared to WT mice (*p* < 0.05) (Figure 1A). Further validation of this finding was obtained through immunohistochemical analysis, which showed that tissue sections obtained from bleomycin-induced fibrotic skin of CD109 KO mice displayed significantly enhanced staining for collagen I as compared to those of WT mice (*p* < 0.0001) (Figure 1B). Additionally, consistent with these findings, Masson’s Trichrome (Figure 1B) and Picrosirius Red staining (Figure 1B) revealed that collagen was more compactly arranged in tissue sections of bleomycin-induced fibrotic skin of CD109 KO mice, when compared to those of WT mice. Moreover, CD109 KO mice display increased bleomycin-induced dermal thickening as compared to WT mice (*p* < 0.0001) (Figure 1B). Finally, CD109 KO mice exhibit epidermal hyperplasia and abnormal epidermal appendages, consistent with findings by [30] (Figure 1C).

**CD109 KO mice display increased dermal ECM production and myofibroblast differentiation as compared to WT mice, during bleomycin-induced skin fibrosis.**

**Figure 2:**
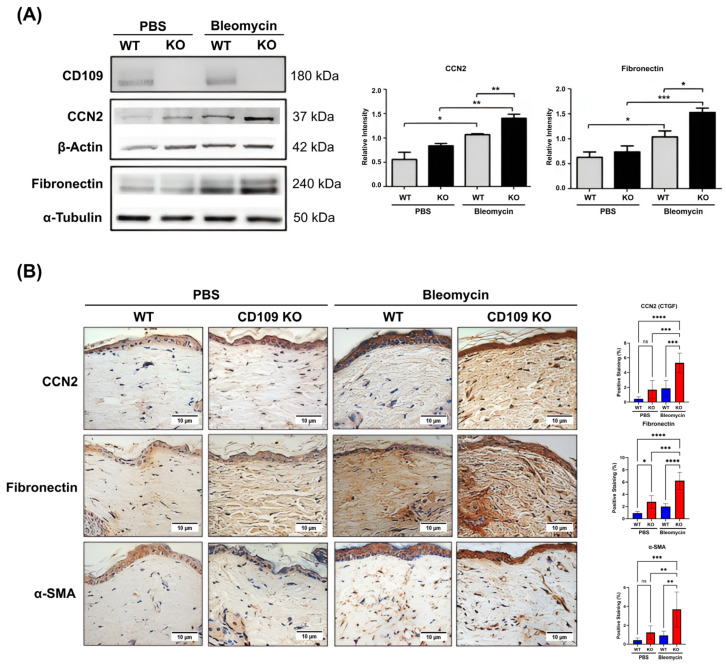
Bleomycin-injected CD109 KO mice displayed increased ECM production and myofibroblast differentiation. WT and CD109 KO mice were treated with PBS or bleomycin injections on alternating days for a period of 28 days (n = 4 per condition). (**A**) Western analysis of CCN2 and fibronectin levels in WT and CD109 KO mice treated with either PBS or bleomycin. Protein expression was quantified using ImageJ and plotted with GraphPad Prism. Student’s *t*-test is used for the calculation of statistical significance. (**B**) Immunohistochemical staining of CCN2, fibronectin, and α-SMA in skin sections from WT and CD109 KO mice treated with PBS or bleomycin. Ordinary one-way ANOVA was used for the calculation of statistical significance. WT: Wild-type, KO: Knockout, PBS: Phosphate-buffered saline, CCN2: Cellular communication network factor 2, α-SMA: Alpha smooth muscle actin. Scale bars: 10 µm. * *p* < 0.05, ** *p* < 0.01, *** *p* < 0.001, **** *p* < 0.0001; ns, not significant.

Our results reveal that during bleomycin-induced skin fibrosis, CD109 KO mice skin exhibits significantly higher levels of fibronectin (*p* < 0.05) and connective tissue growth factor (CTGF, CCN2) (*p* < 0.01) protein expression when compared to WT mice skin, as evidenced by Western blot analysis (Figure 2A). Results from IHC staining for fibronectin (*p* < 0.0001) and CCN2 (*p* < 0.001) provided further validation of those results (Figure 2B). The differentiation of fibroblasts into myofibroblasts is characterized by excessive ECM synthesis and increased α-SMA expression. Our results using IHC staining demonstrate that α-SMA levels are higher in the skin of CD109 KO mice than in WT mice following bleomycin treatment (*p* < 0.01), pointing to increased myofibroblast differentiation in CD109-deficient mice during skin fibrosis (Figure 2B).

**CD109 KO mice display increased phosphorylation of Smad1, Smad2, and Smad3 as compared to WT mice.**

**Figure 3:**
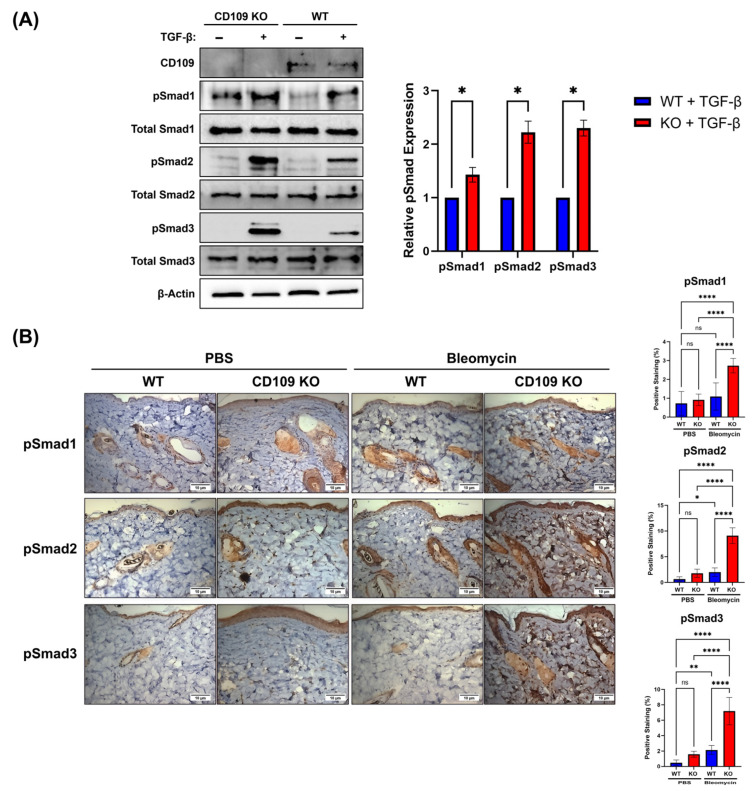
Increased Smad1, Smad2, and Smad3 phosphorylation in CD109 KO mouse dermal fibroblasts and skin. (**A**) Western analysis of pSmad1/2/3 levels in WT and CD109 KO mouse dermal fibroblasts treated with TGF-β (n = 3 each). Protein expression was quantified using ImageJ and plotted with GraphPad Prism 10. Student’s *t*-test is used for the calculation of statistical significance. (**B**) WT and CD109 KO mice received alternating PBS or bleomycin injections for 28 days, after which their skin was analyzed. Immunohistochemical staining of pSmad1, pSmad2, and pSmad3 in skin sections (n = 4 each) from WT and CD109 KO mice treated with PBS or bleomycin. Optical densities were quantified using Image Pro Plus 6 software and plotted using GraphPad Prism. The experiments were statistically tested using an ordinary one-way ANOVA. WT: Wild-type, KO: Knockout, PBS: Phosphate-buffered saline, pSmad1: Phosphorylated Smad1, pSmad2: Phosphorylated Smad2, pSmad3: Phosphorylated Smad3. Scale bars: 10 µm. * *p* < 0.05, ** *p* < 0.01, **** *p* < 0.0001; ns, not significant.

We next investigated the impact of CD109 deletion on activation of Smad2/3 and Smad1 pathways in vitro using dermal fibroblasts treated with TGF-β and in vivo during bleomycin-induced skin fibrosis. Using Western blot and IHC, we examined the levels of phosphorylated Smad1, Smad2, and Smad3 proteins in dermal fibroblasts. Western blot analysis highlights increased pSmad1 (*p* < 0.05), pSmad2 (*p* < 0.05), and pSmad3 (*p* < 0.05) phosphorylation after TGF-β treatment (Figure 3A). IHC analysis revealed elevated levels of pSmad1 (*p* < 0.0001), pSmad2 (*p* < 0.0001), and pSmad3 (*p* < 0.0001) in CD109 KO mice as compared to WT counterparts after bleomycin treatment (Figure 3B).

**CD109 KO fibroblasts showed enhanced TGF-β1-induced cell migration and collagen gel matrix contraction as compared to WT fibroblasts.**

**Figure 4:**
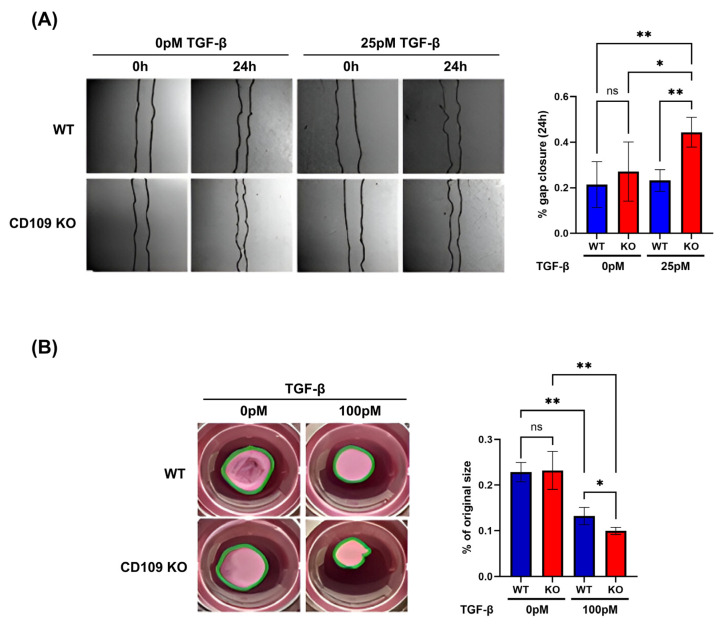
TGF-β-induced wound closure and contraction rates were significantly increased in CD109 KO mouse fibroblasts in vitro. (**A**) Migration/Scratch assay of TGF-β-treated WT and CD109 KO fibroblasts at 0 h and 24 h. The data shown represents at least n = 3 biological replicates with each n = 3 technical replicates. Wound closure was quantified using ImageJ and plotted using GraphPad Prism. (**B**) Collagen matrix contraction test of TGF-β-treated WT and CD109 KO fibroblasts (n = 3). Contraction was quantified using ImageJ and plotted using GraphPad Prism 10. All experiments were statistically tested using an ordinary one-way ANOVA. WT: Wild-type, KO: Knockout, PBS: Phosphate-buffered saline, TGF-β: Transforming growth factor beta. * *p* < 0.05, ** *p* < 0.01, ns, not significant.

Analysis of cell migration using an in vitro wound healing assay (scratch assay) showed that the CD109 KO and WT fibroblasts migrated at similar rates under baseline conditions at 24 h (*p* = 0.698). However, CD109 KO fibroblasts showed a significantly higher rate of TGF-β1-induced cell migration as compared to WT fibroblasts (*p* < 0.01) (Figure 4A). Stimulation with 25 pM TGF-β1 of CD109 KO fibroblasts showed a wound gap closure of 44.35 ± 6.5% at 24 h versus 23.16 ± 4.8% in WT fibroblasts (*p* < 0.01) (Figure 4A). In addition, CD109 KO fibroblasts showed a significantly greater increase in TGF-β1-induced collagen gel contraction as compared to WT fibroblasts (*p* < 0.05) (Figure 4B).

**Figure 5:**
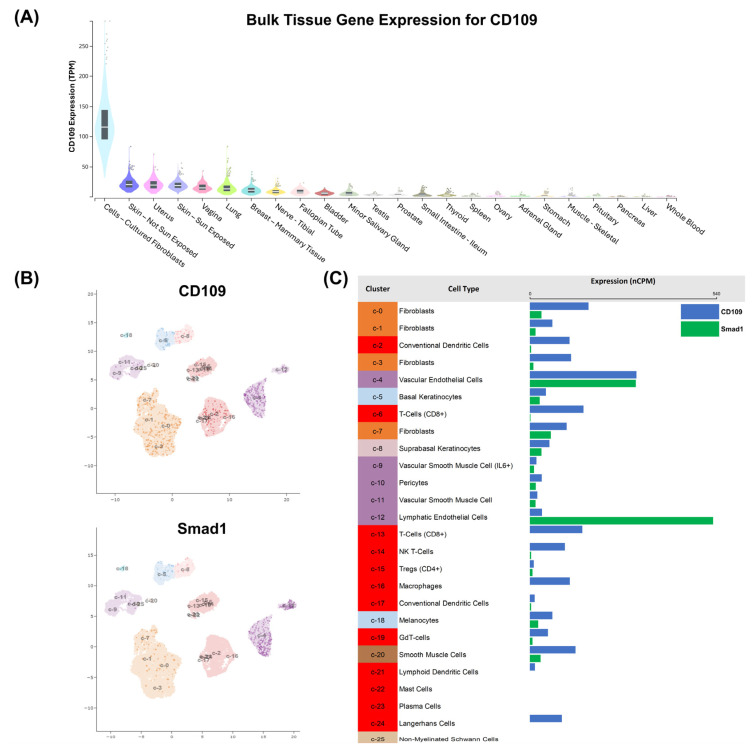
CD109 is widely expressed in human tissues in the body (**A**) and is differentially expressed in subpopulations of cells in the skin ((**B**), upper panel, and (**C**)), with the expression of CD109 inversely correlating with that of Smad1 in fibroblasts ((**B**), lower panel, and (**C**)). (**A**) Bulk tissue RNA expression of CD109 across human tissues from the GTEx Analysis Release V10, shown as transcripts per million (TPM). (**B**) Single-cell RNA expression of CD109 and Smad1, visualized by a UMAP (see methods). (**C**) Side-by-side comparison of CD109 and Smad1 expression levels across cell clusters, shown in normalized counts per million (nCPM). The colors of each cluster name correspond to the colors on the UMAP in (**B**).

**CD109 is widely expressed in human tissues (Figure 5A) and it is differentially expressed in subpopulations of skin-associated cells (Figure 5B upper panel and Figure 5C).**

To examine CD109 expression in the human body, we analyzed publicly available bulk tissue RNA expression data from the Genotype-Tissue Expression Project (GTEx) database, which contains measurements from many different human tissues. CD109 expression, reported as transcripts per million (TPM), was compared across a wide range of tissues using visualization tools available on the GTEx Portal.

Bulk transcriptomic analysis demonstrated that CD109 is highly expressed in fibroblast-rich tissues, including skin, while showing minimal expression in most internal organs and circulating blood, indicating preferential enrichment within mesenchymal compartments (Figure 5A). To further explore gene expression at the single-cell level in human skin, we used single-cell RNA sequencing datasets from the Human Protein Atlas single-cell database to identify gene expression in individual skin cell populations. Expression levels for CD109 and Smad1 were summarized as normalized counts per million (nCPM) and visualized as bar charts corresponding to clusters of cells identified by single-cell RNA sequencing analysis and mapped using Uniform Manifold Approximation and Projection (UMAP) plots. These results revealed that CD109 expression is enriched in fibroblast clusters and vascular endothelial cells (Figure 5B,C). In contrast, Smad1 displayed a broader distribution but was reduced in CD109-high fibroblast clusters, showing an inverse correlation with CD109 expression, while higher levels were observed in endothelial and lymphatic endothelial cells (Figure 5B,C).

## 3. Discussion

Skin fibrosis, characterized by excessive ECM deposition and tissue remodeling, is the hallmark of conditions such as hypertrophic scarring and keloid formation, where wound healing is dysregulated [1], and of diseases like scleroderma (systemic sclerosis, SSc), where fibrosis extends beyond the skin to internal organs [2]. We have shown previously that CD109 protein levels are increased in SSc patient skin as compared to normal healthy skin [29] and decreased in psoriasis patient skin as compared to adjacent uninvolved skin [32], suggesting that dysregulation of CD109 expression may play a pathological role in these conditions. We have also shown that TG mice overexpressing CD109 in the epidermis show improvement in wound healing and fibrosis parameters in experimental models in vivo [27,28]. Altering CD109 expression in the dermal fibroblasts has not been investigated. In the current study, using a global CD109 KO mouse model, we show that CD109-deficient mice exhibit enhanced bleomycin-induced skin fibrosis, as characterized by increased dermal thickening, ECM production and myofibroblast differentiation when compared to WT mice. Although collagen I levels did not differ significantly between WT and CD109 KO skin under basal conditions, Western blot analysis revealed a consistent trend toward increased collagen I in CD109-deficient mice. While not statistically significant, this observation raises the possibility that CD109 contributes to the regulation of basal dermal collagen homeostasis. Subtle alterations in ECM set-points under resting conditions may predispose tissue to exaggerated fibrotic responses upon injury or challenge, and future studies will be required to directly address this possibility. This enhanced skin fibrosis is associated with heightened activation of Smad1, Smad2, and Smad3 signaling, confirming that CD109 functions as a negative regulator of TGF-β-driven fibrotic responses in the skin. Additionally, isolated CD109 KO mouse fibroblasts demonstrate increased TGF-β1-induced migration and collagen gel matrix contraction, further underscoring the role of endogenous CD109 in controlling fibroblast activity and fibrosis progression.

CD109 expression is elevated in scleroderma skin, likely reflecting a compensatory response to chronic TGF-β activation, but reduced in psoriatic lesions, where loss of CD109-mediated inhibition of TGF-β contributes to keratinocyte hyperproliferation and aberrant ECM remodeling [29,32,36]. These opposing patterns suggest distinct, cell-type-specific roles for CD109, restraining TGF-β-driven fibrosis in dermal fibroblasts and maintaining epidermal homeostasis in keratinocytes [37]. Further, understanding how CD109 interacts with immune and endothelial cells within the dermal microenvironment may reveal additional mechanisms underlying its anti-fibrotic activity in skin [38].

While our finding that CD109 deletion leads to increased phosphorylation of Smad2 and Smad3 proteins in the current study is consistent with our previous report that epidermal overexpression of CD109 diminishes phosphorylation of Smad2 and Smad3 [24], our observation that Smad1 phosphorylation is upregulated in both the CD109 deletion (current study) and overexpression [24] models is intriguing. The analysis of the gene bulk expression and single-cell databases demonstrated an inverse expression pattern in skin fibroblast cell populations with elevated CD109 expression associated with lower Smad1 expression (Figure 5). This inverse relationship supports the concept that CD109-rich fibroblast populations are associated with reduced Smad1 transcriptional output, consistent with a modulatory role for CD109 in shaping Smad-dependent signaling landscapes within mesenchymal cells relevant to fibrotic regulation. However, the possibility that the increased Smad 1 phosphorylation is bone morphogenetic protein (BMP)-mediated (and not TGF-β-induced) cannot be ruled out. The critical role of Smad3 pathway activation in mediating fibrosis is well established. For example, targeting Smad3 can attenuate fibrosis [39], and disrupting Smad3 signaling reduces collagen synthesis, accumulation, and transcription, leading to less fibrosis [40]. The role of Smad2 and Smad1 in mediating skin fibrosis is less well understood. The relative significance of Smad3, Smad2, Smad1 and MAP kinases in mediating the antifibrotic effects of CD109 is an interesting question to pursue.

While the present study focused on defined components of the TGF-β signaling pathway and established fibrotic markers, the CD109 KO mouse model offers a powerful platform for broader discovery-based analyses. Unbiased approaches such as global transcriptomic or proteomic profiling of WT and CD109-deficient skin, both at baseline and following fibrotic challenge, may reveal additional signaling pathways, regulatory networks, and previously unrecognized mediators contributing to altered skin homeostasis and fibrosis susceptibility. Such studies could further clarify cell-type-specific and non-canonical mechanisms regulated by CD109 and expand our understanding of its role beyond the classical TGF-β axis.

A limitation of this study is the use of the bleomycin-induced mouse model, which, although widely used [39], does not fully reflect the complexity of human fibrotic skin disorders such as scleroderma [41]. In addition, the use of a global CD109 KO mouse model precludes cell-type-specific interpretation of the observed effects. Consequently, potential contributions from non-fibroblast populations cannot be excluded, and these may have influenced the fibrotic phenotype independently of fibroblast-intrinsic mechanisms. A further limitation is that we did not directly quantify TGF-β receptor protein levels (e.g., TβRII/ALK5/ALK1) in WT vs. CD109 KO skin; however, our pathway readouts (pSmads) and fibrosis endpoints robustly indicate heightened signaling in CD109-deficient skin. Furthermore, in previous studies, we have demonstrated that CD109 is an important regulator of TGF-β receptor levels [30,31,32]. Future studies using fibroblast-specific CD109 knockout mice and patient-derived skin biopsies will clarify CD109’s role in fibrosis and inflammation. Integrating genetic models with human data will better define their cell-specific functions and therapeutic potential in fibrotic skin disease.

In summary, this study identifies CD109 as a critical negative regulator of dermal fibrosis. Loss of CD109 enhanced fibrotic responses in vivo, as reflected by increased ECM deposition, myofibroblast differentiation, and Smad2/3 activation in bleomycin-treated CD109 KO mice relative to wild-type controls. Consistently, CD109-deficient fibroblasts displayed enhanced migration and collagen contraction in vitro, indicative of a profibrotic phenotype. Collectively, these findings position CD109 as a key regulator of skin homeostasis and underscore its potential as a therapeutic target in fibrotic skin disease.

## 4. Materials and Methods

**Generation of Mouse Colony:** Frozen sperm from CD109 heterozygotic C57BL/6 mice was obtained from Dr. Masahide (Department of Pathology, Nagoya University Graduate School of Medicine, Nagoya, Japan) [33] and used to expand the colony. For experiments, sixteen CD109 KO and sixteen WT male mice, aged 4–6 weeks, were selected from third-generation offspring.

**Genotyping of CD109 Mice:** Genomic DNA was extracted from mouse tail samples using the REDExtract-N-Amp™ Tissue PCR Kit (XNAT, Sigma-Aldrich, St. Louis, MO, USA). PCR was performed with specific primers to identify CD109 KO and WT alleles. Genotyping of mice was conducted using PCR with four specific primers: P1 (forward) 5′-GTCCCGCTTTCTGGTGACAG-3′, P2 (reverse) 5′-GTGTGACTGTTAGACAGTGCAG-3′, P3 (forward) 5′-CCATCGCCATCTGCTGCACG-3′, and P4 (reverse) 5′-ACGATCCTGAGACTTCCACAC-3′. PCR amplification was carried out under the following conditions: initial denaturation at 96 °C for 2 min, followed by 32 cycles of denaturation at 94 °C for 30 s, annealing at 65 °C for 30 s, and extension at 72 °C for 30 s. The expected amplicon sizes were 205 bp for the wild-type allele and 603 bp for the targeted allele [33].

**In Vivo Bleomycin Treatment:** Thirty-two mice, comprising sixteen CD109 KO and sixteen WT mice, were divided into groups receiving either bleomycin dissolved in phosphate-buffered saline (PBS) or PBS alone. Under anesthesia with isoflurane, their dorsal skin was shaved, and a 1 × 1 cm area for injection was outlined. The mice were subjected to treatment every other day, receiving either a 100 μL subcutaneous injection at a single site of bleomycin sulfate (15 μg, Wisent Bioproducts) in PBS or an equivalent volume of PBS alone. This regimen was maintained over a span of 28 days. Mice were euthanized on day 29 for sample collection.

**Histology and Immunohistochemistry (IHC):** Treated skin areas were processed for Hematoxylin and Eosin, Masson’s trichrome, or Picrosirius Red staining. Dermal thickness was quantified from the basement membrane to the hypodermis across five high-power fields for each section, with two distinct sections evaluated per specimen. This analysis was conducted using Image Pro Plus 6 software (Media Cybernetics, Bethesda, MD, USA). IHC was carried out at 4 °C overnight using specific antibodies to evaluate the expression levels of fibrosis markers such as α-smooth muscle actin (α-SMA), type I collagen, connective tissue growth factor (CTGF, CCN2), and fibronectin, compared to IgG negative controls. Additionally, the activation of TGF-β downstream signaling proteins, including phospho-Smad1 (pSmad1), phospho-Smad2 (pSmad2), and phospho-Smad3 (pSmad3), was assessed in a similar manner. The quantification of these markers was achieved by measuring the optical density of the calibrated images, which were restricted to the fibroblast-rich dermal compartment, using Fiji ImageJ 1.54g software [42]. The statistical significance was calculated using an ordinary one-way ANOVA.

**Western Blot and Densitometry:** Full-thickness skin tissue was homogenized in RIPA buffer supplemented with protease inhibitor cocktail, sodium orthovanadate and phenylmethylsulphonyl fluoride (PMSF). Tissue homogenate was clarified at 12,000× *g* for 10 min. The supernatant was collected for protein quantification using the Bio-Rad Lowry Protein Assay (Bio-Rad Laboratories, Hercules, CA, USA). Samples were resolved by 7.5% or 10% SDS-PAGE and transferred to a nitrocellulose membrane. Measurement of markers of fibrosis (α-SMA, fibronectin, CTGF, collagen I) was performed using primary and HRP-conjugated secondary antibodies. Each experiment was performed in at least quadruplicate, and densitometry of immunoblots was measured using ImageJ 1.54g software (NIH) [43]. Student’s *t*-test was used for the calculation of statistical significance.

**Isolation of Dermal Fibroblasts:** CD109 KO mice and their WT mice, aged 0 to 5 days, were euthanized, the treated skin area was collected and fibroblasts were isolated. The procedure involved cleaning the bodies with 10% Poviodine-Iodine solution (Laboratoire Atlas Inc., Montreal, QC, Canada), rinsing with sterile water, and harvesting the skin under sterile conditions. The skin was minced and digested in a 0.1% collagenase-containing medium. After incubation and stirring for up to 2 h, the mixture was centrifuged at 180× *g* for 10 min to pellet the cells, which were then washed and suspended in fresh medium (DMEM). The cell culture was maintained at 37 °C with 5% CO_2_, with a medium change after 24 h and subsequently as needed.

**Fibroblast Cell Count and Proliferation:** Mouse fibroblasts were prepared at a concentration of approximately 1 × 10^6^ cells/mL. For viability assessment, cells were stained using a 0.4% trypan blue solution and allowed to sit for 5 min at room temperature. A 20 μL sample of the cell-trypan blue mixture was then applied to a hemocytometer to count both viable and non-viable cells. In total, 0.3 × 10^5^ CD109 KO and WT fibroblasts were seeded in each well of six-well plates and cultured under previously described conditions for dermal fibroblast isolation. Upon reaching confluence, fibroblasts were detached and recounted using the trypan blue exclusion method to compare viable cell counts between KO and WT cell populations.

**In Vitro Wound Healing Scratch Assay:** Third-passage CD109 KO and WT mouse fibroblasts (0.1 × 10^6^ cells) were cultured to confluence, serum-starved for 6.5 h, and then treated with mitomycin C (to inhibit proliferation) and with 0 pM, 25 pM, or 100 pM of TGF-β1 (Sanofi Genzyme), respectively. A scratch assay was performed on the cell monolayer using a 200 μL pipette tip, and gap closure was monitored over 24 h using serial photography. The extent of cell migration was quantified using ImageJ 1.54 g software [43] based on the closure of the scratched area. The statistical significance was calculated using an ordinary one-way ANOVA.

**Collagen Contraction Assay:** A total of 10 mg of rat tail collagen (11179179001, Sigma-Aldrich) was dissolved in 2.5 mL of 0.2% mM acetic acid solution at 4 °C, according to the manufacturer’s protocol, for a final concentration of 4 mg/mL. This collagen solution was mixed with DMEM containing 10% fetal bovine serum, NaOH and third passage CD109 KO and WT mouse fibroblasts to create a uniform gel mixture. This mixture was aliquoted into a 24-well plate and allowed to solidify at room temperature for 30 min. After solidification, 800 μL of supplemented medium was added, and the plate was incubated at 37 °C with 5% CO_2_ overnight. Post-incubation, fibroblasts were serum-starved for 6–8 h and treated with TGF-β1. Photographic documentation was made at 0 and 24 h to assess collagen contraction, which was quantified using ImageJ 1.54g software (NIH) [43] based on changes in gel size. The statistical significance was calculated using an ordinary one-way ANOVA.

**Bulk Tissue Gene Expression and Single-Cell RNA Sequencing Analysis:** Bulk tissue RNA expression data for CD109 (ENSG00000156535.15) were obtained from the Genotype-Tissue Expression (GTEx) Project, Analysis Release V10 (dbGaP accession phs000424.v10.p2), and reported as transcripts per million (TPM) across a broad range of human tissues using GTEx-provided visualization tools (https://www.gtexportal.org/home/gene/CD109/geneExpressionTab; accessed on 21 January 2026). Single-cell RNA sequencing data for CD109 and Smad1 were obtained from the Human Protein Atlas (HPA) (https://www.proteinatlas.org/; accessed 21 January 2026) single-cell database, which integrates curated human single-cell transcriptomic datasets. Cluster-level gene expression was summarized using normalized counts per million (nCPM) and visualized as bar charts linked to the corresponding UMAP clusters.

## Data Availability

The data presented in this study is available on request from the corresponding author. Publicly available datasets analyzed in this study can be accessed through the GTEx Project (https://www.gtexportal.org/home/gene/CD109/geneExpressionTab, accessed on 3 December 2025) and the HPA (https://www.proteinatlas.org/, accessed on 3 December 2025). Experimental data generated in this study are not publicly available due to institutional and ethical restrictions but are available from the corresponding author upon reasonable request.

## Abbreviations

The following abbreviations are used in this manuscript:

CD109	Cluster of Differentiation 109
SSc	Systemic Sclerosis
TGF-β	Transforming Growth Factor Beta
ECM	Extracellular Matrix
IHC	Immunohistochemistry
WT	Wild-type
KO	Knockout
